# Short-term early exposure to lapatinib confers lifelong protection from mammary tumor development in MMTV-erbB-2 transgenic mice

**DOI:** 10.1186/s13046-016-0479-8

**Published:** 2017-01-06

**Authors:** Zhikun Ma, Amanda B. Parris, Zhengzheng Xiao, Erin W. Howard, Stanley D. Kosanke, Xiaoshan Feng, Xiaohe Yang

**Affiliations:** 1 Julius L. Chambers Biomedical/Biotechnology Research Institute, Department of Biological and Biomedical Sciences, North Carolina Central University, 500 Laureate Way, Room 4301, Kannapolis, NC 28081 USA; 2 Department of Oncology, First Affiliated Hospital of Henan University of Sciences and Technology, Luoyang, China; 3 Department of Pathology, University of Oklahoma Health Sciences Center, Oklahoma City, OK 73104 USA

**Keywords:** Crosstalk, EGFR, ErbB-2/Her2, Estrogen receptor (ER), Lapatinib, MMTV-erbB-2 transgenic mice

## Abstract

**Background:**

Although chemopreventative agents targeting the estrogen/estrogen receptor (ER) pathway have been effective for ER^+^ breast cancers, prevention of hormone receptor-negative breast cancers, such as Her2/erbB-2^+^ breast cancers, remains a significant issue. Previous studies have demonstrated that administration of EGFR/erbB-2-targeting lapatinib to MMTV-erbB-2 transgenic mice inhibited mammary tumor development. The prevention, however, was achieved by prolonged high dose exposure. The tolerance to high dose/long-term drug administration may hinder its potential in clinical settings. Therefore, we aimed to test a novel, short-term chemopreventative strategy using lapatinib during the premalignant risk window in MMTV-erbB-2 mice.

**Methods:**

We initially treated cultured cells with lapatinib to explore the anti-proliferative effects of lapatinib in vitro. We used a syngeneic tumor graft model to begin exploring the in vivo anti-tumorigenic effects of lapatinib in MMTV-erbB-2 mice. Then, we tested the efficacy of brief exposure to lapatinib (100 mg/kg/day for 8 weeks), beginning at 16 weeks of age, in the prevention of mammary tumor development in MMTV-erbB-2 mice.

**Results:**

In the syngeneic tumor transplant model, we determined that lapatinib significantly inhibited tumor cell proliferation. Furthermore, we demonstrated that short-term lapatinib exposure resulted in life-long protective effects, as supported by increased tumor latency in lapatinib-treated mice compared to the control mice. We further established that delayed tumor development in the treated mice was preceded by decreased BrdU nuclear incorporation and inhibited mammary morphogenesis. Molecular analysis indicated that lapatinib inhibited phosphorylation and expression of EGFR, erbB-3, erbB-2, Akt1, and Erk1/2 in premalignant mammary tissues. Also, lapatinib drastically inhibited the phosphorylation and expression of ERα and the transcription of ER target genes in premalignant mammary tissues. We also determined that lapatinib suppressed the stemness of breast cancer cell lines, as evidenced by decreased tumorsphere formation and ALDH^+^ cell populations.

**Conclusions:**

Taken together, these data demonstrate that brief treatment with EGFR/erbB-2-targeting agents before the onset of tumors may provide lifelong protection from mammary tumors, through the concurrent inhibition of erbB-2 and ER signaling pathways and consequential reprogramming. Our findings support further clinical testing to explore the benefit of shorter lapatinib exposure in the prevention of erbB-2-mediated carcinogenesis.

## Background

Although early diagnosis and various therapies for breast cancer patients have improved clinical outcomes, reducing the prevalence of breast cancer risk and eradicating this morbid disease remains a significant challenge. Therefore, development of novel preventive agents and strategies is of pivotal importance in this regard. For breast cancer prevention, use of endocrine modulators that target estrogen signaling or production, such as tamoxifen or letrozole, has proven to be a successful strategy to prevent and treat estrogen receptor-positive (ER^+^) breast cancers [1–3]. However, effective management of ER-negative (ER-) breast cancers remains under development.

erbB-2/Her2^+^ breast cancers account for approximately 30% of breast cancer cases [4]. Likewise, erbB-2 amplification has been associated with poor prognosis, metastasis, and therapeutic resistance [5, 6]. erbB-2 is a member of the epidermal growth factor receptor (EGFR) family, which also includes EGFR, erbB-3, and erbB-4. This family of receptor tyrosine kinases (RTKs) plays a critical role in cell proliferation, survival, migration, and angiogenesis [7–9]. Of note, erbB-2 is an ‘orphan receptor’ and its activation mainly relies on its interaction with other EGFR family members [10–12]. A number of novel therapeutics targeting erbB-2 and its family members have been developed and used clinically, which has significantly improved patient outcomes [13, 14]. Of these novel agents, lapatinib, an oral RTK inhibitor, has been approved by the US Food and Drug Administration for use on erbB-2^+^, locally advanced or metastatic breast cancers [15]. Lapatinib reversibly binds to the kinase domains of erbB-2 and EGFR to block receptor phosphorylation and activation, which results in the inhibition of downstream signaling pathways, including the mitogen-activated protein kinase (MAPK)/Erk and phosphatidylinositol 3-kinase (PI3K)/Akt pathways [16–18]. Selective inhibition of erbB-2/EGFR-mediated signaling presents lapatinib as a promising drug to target erbB-2/EGFR-overexpressing breast cancers.

While the development of erbB-2-targeted therapeutics continues, preventative strategies targeting erbB-2/EGFR are emerging. It has been demonstrated that lapatinib prevents mammary tumor development in mouse mammary tumor virus (MMTV)-erbB-2 transgenic mice; however, the chemopreventive effect in the study was based on long-term, high dose exposure to lapatinib (12 months, 75 mg/kg twice daily) [19]. Although this drug is regarded as well-tolerated, life-long treatment may compromise the application of lapatinib as a chemopreventive agent. In order to further unveil the possibility of lapatinib therapy in breast cancer chemoprevention, we investigated the effect of short-term lapatinib exposure during the premalignant risk window on the development of mammary tumors in MMTV-erbB-2 transgenic mice. MMTV-erbB-2 transgenic mice are a clinically relevant model of erbB-2-overexpressing breast cancers with a defined genetic background and tumors forming at approximately 35 weeks of age. Our strategy aimed to use lapatinib to prevent erbB-2-mediated carcinogenesis with a short-term, low dose (8 weeks, 100 mg/kg once daily) approach to demonstrate a lesser risk of toxicity, better medication adherence, and, thereby, an acceptable risk-benefit ratio. All of these factors are critical to the success of a cancer risk reduction intervention in clinical practice. Indeed, we found that this strategy can delay the development of mammary tumors in MMTV-erbB-2 transgenic mice.

## Methods

### Reagent and antibodies

Lapatinib was purchased from LC Laboratories (Woburn, MA). The following antibodies were purchased from Cell Signaling (Danvers, MA): EGFR, phospho-EGFR (Ser1046/1047), phospho-erbB-2 (Tyr877), phospho-erbB-3 (Tyr1289), phospho-Akt (Ser473), and phospho-ERα (Ser167); Millipore (Temecula, CA): ERα and erbB-2; and Santa Cruz Biotechnology (Santa Cruz, CA): erbB-3, c-Myc, Erk2, phospho-Erk, Bcl-2, cyclin D1, and β-actin. Horseradish peroxidase (HRP)-labeled goat anti-rabbit and anti-mouse secondary antibodies were used from Thermo Scientific (Rockford, IL).

### Cell culture

The erbB-2-overexpressing mammary tumor cell lines, 78617 and 85815, used in this study were established from mammary tumors of FVB/N-Tg/MMTV-erbB-2 mice as previously described [20]. BT474 and SKBR3 cells were purchased from the American Type Culture Collection (ATCC; Manassas, VA). All cells were maintained in DMEM/F-12 culture medium supplemented with 10% fetal bovine serum (FBS), penicillin (100 μg/ml), and streptomycin (100 μg/ml) in a humidified incubator with 5% CO_2_ at 37 °C.

### MTS assay

In vitro cell proliferation was determined by an MTS assay using the CellTiter 96 AQueous One Solution Cell Proliferation assay kit (Promega; Madison, WI) according to the manufacturer’s instructions. Briefly, 500 cells per well were seeded in 96-well plates. After 24 h, cells were treated with indicated concentrations of lapatinib for 4 days. Then, MTS solution was added to each well and incubated for 2 h at 37 °C. The survival fraction was determined based on the absorbance detected at 490 nm using the SynergyMx microplate reader (BioTek; Winooski, VT). Each experiment was performed in triplicate.

### Western blotting

Cells or homogenized tissues were incubated in lysis buffer to extract total protein. The protein was quantified using a BCA Protein Assay kit (Thermo Scientific Pierce). Equal sample concentrations (50 μg protein) were loaded in 10 or 12% SDS-PAGE gels for electrophoresis. After separation, proteins were transferred to nitrocellulose membranes and blocked in 5% non-fat dry milk for 1 h. Primary antibodies were incubated overnight at 4 °C. Then, membranes were washed and incubated in appropriate HRP-labeled secondary antibodies for 1.5 h at room temperature. Proteins were enhanced using SuperSignal West Pico Chemiluminescent solution (Thermo Fisher Scientific) and detected using a FluorChemE imager.

### Animals and treatments

Female FVB/N-Tg/MMTV-erbB-2 (MMTV-erbB-2) transgenic mice were purchased from Jackson Labs (Bar Harbor, ME). The mice were fed an AIN-93G diet (Harlan Teklad; Madison, WI). All animal experiments were conducted according to IACUC-approved protocols.

For the syngeneic tumor graft model, viable 78617 cells (1 × 10^6^) were subcutaneously injected into the flanks of 8-week-old mice (*n* = 5 per group). After 7 days, lapatinib (100 mg/kg) or vehicle (distilled water containing 1% Tween 80) were administered daily via oral gavage for 14 days. Tumor volumes were calculated based on the formula: tumor volume = longest diameter × shortest diameter^2^ × 0.5. By the endpoint, mice were euthanized and tumors were collected for analysis.

For the preventive experiment using a short-term lapatinib treatment, MMTV-erbB-2 transgenic mice were administered vehicle or lapatinib (100 mg/kg/day) via oral gavage 6 days a week from 16 weeks of age to 24 weeks of age (*n* = 30 per group). After 8 weeks of treatment (at 24 weeks of age), five mice from each group were euthanized and mammary glands were collected for whole mount, immunohistochemistry (IHC), protein, and RNA analyses. The remaining mice in each group were monitored for tumor development by twice-a-week examination beginning at 20 weeks of age. The latency of tumor development was defined as the detection of the first palpable tumor. The tumor-free interval was presented with a Kaplan-Meier survival curve.

### Mammary whole mount preparation

Tissue from the abdominal mammary gland was mounted onto a glass slide and fixed in Carnoy’s solution (6:3:1 ratio of 100% ethanol:chloroform:glacial acetic acid). Following overnight fixation, samples were rehydrated in serial dilutions of ethanol for 30 min and were stained overnight in carmine alum stain. Then, the samples were dehydrated with 70, 95, and 100% ethanol before being cleared in xylene and mounted with Permount (Thermo Fisher Scientific). The ductal architecture of whole mounts was examined under the Nikon Eclipse 80i microscope and images were captured using the Nikon Elements Imaging System (Nikon Instruments, Inc.).

### BrdU incorporation assay

Mice were intraperitoneally injected with 200 μl of 5-bromo-2’-deoxyuridine (BrdU; 3 mg/ml) solution 90 min before euthanization. The inguinal mammary glands were collected and fixed with 10% formalin for paraffin embedding, followed by BrdU detection with IHC.

### Terminal deoxynucleotidyl transferase-mediated dUTP nick end labeling (TUNEL) assay

Apoptosis was measured in tumor and mammary gland tissues using an ApopTag Peroxidase In Situ Apoptosis Detection kit (Millipore) according to the manufacturer’s instructions. Briefly, formalin-fixed paraffin-embedded (FFPE) tissue sections (5 μm thick) were deparaffinized with xylene and rehydrated with ethanol. Then, proteinase K (20 μg/ml) was added to the slides at room temperature for 15 min. After washing in deionized water, endogenous peroxidase activity was blocked with 3% H_2_O_2_ at room temperature for 5 min. Slides were then washed in PBS and incubated in Equilibration Buffer, followed by 1 h incubation in TdT enzyme at 37 °C. The reaction was stopped by adding Stop/Wash Buffer at room temperature for 10 min. Next, anti-digoxignenin conjugate was added to each slide and the slides were incubated at room temperature for 30 min. The slides were washed four times in PBS before the color was developed using diaminobenzidine (DAB) at room temperature. The slides were immediately washed in deionized water to stop the DAB reaction, counterstained with hematoxylin, dehydrated with xylene, and mounted with Permount. Stained sections were analyzed using the Nikon Eclipse 80i microscope and Nikon Elements Imaging System Software. The number of tumor or mammary epithelial cells with TUNEL-specific staining was recorded to calculate the percentage of TUNEL-positive cells.

### Immunohistochemistry

FFPE mammary gland sections were deparaffinized and rehydrated as mentioned above in the TUNEL assay protocol. Antigen retrieval was performed by boiling tissue sections in citrate buffer (pH 6.0) at 100 °C for 30 min, followed by DNA denaturation with 2 N HCl at 37 °C for 30 min for the BrdU-treated samples. Next, endogenous peroxidase activity was blocked with 3% H_2_O_2_ in methanol for 10 min at room temperature. Nonspecific binding was then blocked with 10% horse serum prior to overnight incubation at 4 °C with primary antibodies against BrdU (diluted 1:1000) and ERα (diluted 1:2000). The sections were incubated with appropriate secondary antibodies for 1 h, followed by exposure to the ABC reagent (Vector Laboratories; Burlingame, CA) and DAB. Slides were counterstained with hematoxylin and mounted for observation. Stained sections were analyzed using the Nikon Eclipse 80i microscope and Nikon Elements Imaging System Software. The number of mammary epithelial or tumor cells with specific staining of BrdU or ERα was recorded to calculate the percentage of positive cells.

### Real-time PCR

Total RNA was isolated from mammary tissues using a Qiagen RNeasy Mini kit (Qiagen; Valencia, CA) according to the manufacturer’s recommendations. RNA (1 μg) was reversely transcribed using iScript cDNA Synthesis Kit (Bio-Rad; Hercules, CA). Real-time PCR (RT-PCR) was performed using a CFX 96TM Real-Time PCR System (Bio-Rad). The PCR reactions were initiated with enzyme activation at 95 °C for 30 s, followed by 40 amplification cycles at 95 °C for 5 s and 56–60 °C for 10 s. Melt curves were completed at 65–95 °C for 10 s per step. Samples were analyzed in triplicate and β-actin was used for normalization. The relative mRNA levels of each gene in the lapatinib-treated samples versus the control samples were calculated using the comparative Ct (2^ΔΔCt^) method.

### Tumorsphere assay

BT474 or 78617 cells were seeded (2 × 10^3^ cells/well) in triplicate in ultra-low attachment 6-well plates (Corning). Spheres were treated with indicated doses of lapatinib and incubated in DMEM/F-12 medium supplemented with 5 mg/ml insulin (Sigma; St. Louis, MO), 0.5 μg/ml hydrocortisone (Sigma), 1x B-27 (Thermo Fisher Scientific), 20 ng/ml EGF (Sigma), 20 ng/ml bFGF (Stemcell Technologies; Cambridge, MA), and 4 μg/ml heparin (Stemcell Technologies) for 6 days. The number of primary spheres were counted based on sphere sizes ranging from 80 to 120 μm in diameter. Then, primary spheres were trypsinized and vigorously pipetted to form a single cell suspension. To analyze secondary sphere formation, these collected cells were resuspended in the sphere culture medium under the given conditions for another 6 days. The recorded primary and secondary tumorsphere numbers were graphed for comparison.

### ALDH1 assay

Flow cytometric analysis of aldehyde dehydrogenase 1 (ALDH1) activity was measured using an ALDEFLUOR Kit (Stemcell Technologies) and Cell Lab Quanta SC software (Beckman Coulter) according to the manufacturers’ instructions. Briefly, control or lapatinib-treated cells were incubated with the ALDEFLUOR substrate for 30 min at 37 °C. The specific inhibitor of ALDH, diethylaminobenzaldehyde (DEAB), was used to establish the baseline fluorescence and define the ALDEFLUOR-positive region. All treatments were performed and analyzed by flow cytometry in triplicate.

### Statistical analysis

The tumor latency differences between the groups were examined using a log rank test based on Kaplan-Meier survival curves. The significant differences between two groups in all other experiments were determined by two-sided Student’s t-tests. *P*-values of ≤ 0.05 were considered statistically significant.

## Results

### Lapatinib suppresses cell proliferation of 78617 and 85815 cells in vitro through inhibition of RTK signaling

**Fig. 1:**
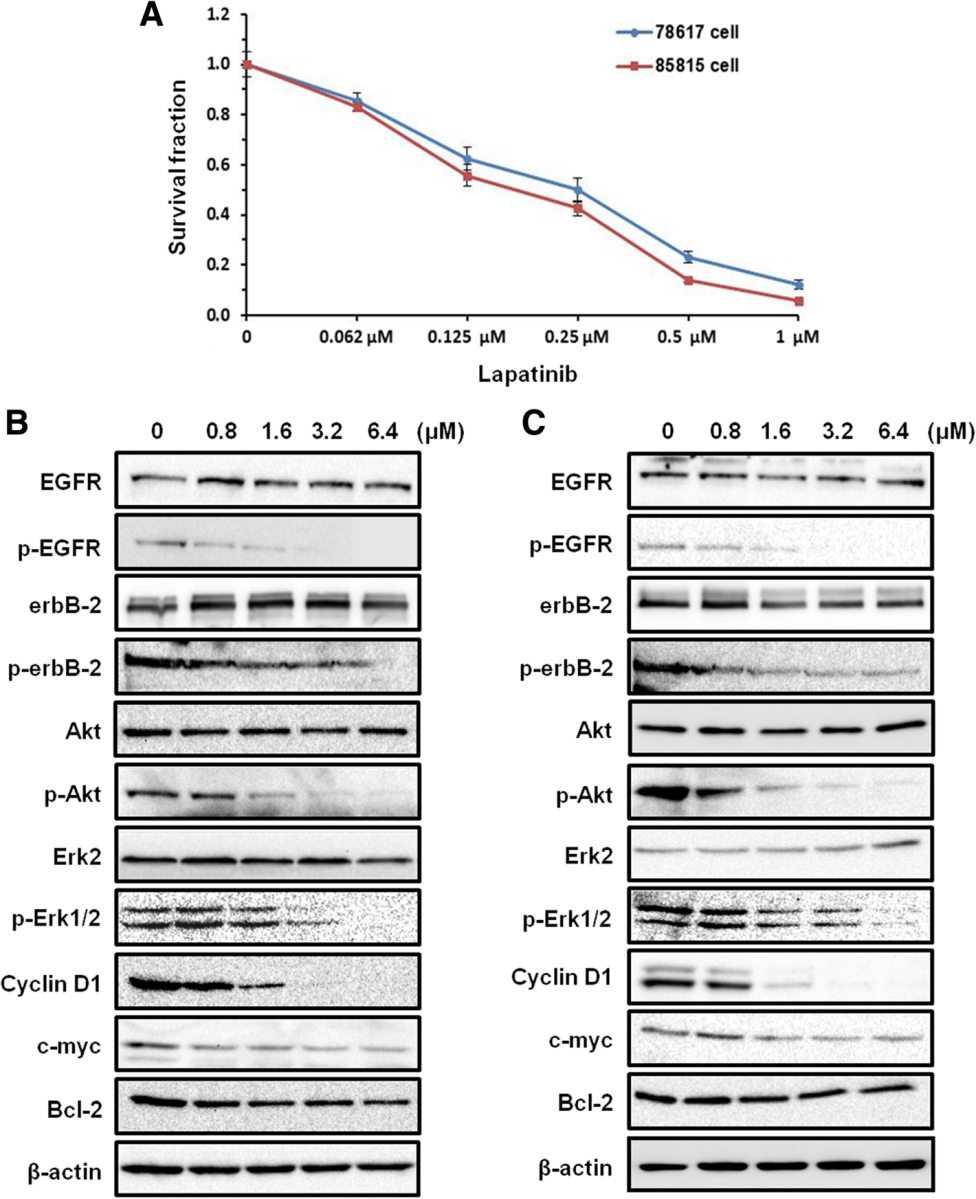
Lapatinib suppresses cell proliferation of 78617 and 85815 cells in vitro through RTK signaling inhibition. **a** An MTS assay of cell proliferation in 78617 and 85815 cells is shown after exposure for 4 days of indicated concentrations of lapatinib. Values are displayed as means ± standard error (S.E.). 78617 (**b**) and 85815 (**c**) cells were treated with lapatinib at indicated concentrations for 24 h. Expression and phosphorylation of specific markers were detected using Western blot analysis

As a preliminary experiment for in vivo studies, we tested the effects of lapatinib on cell proliferation in erbB-2-overexpressing 78617 and 85815 cells, which were derived from MMTV-erbB-2 transgenic mouse mammary tumors [20]. As in Fig. 1a, we showed that exposure to low dose lapatinib (0.062 – 1 μM) dose-dependently decreased cell survival in both cell lines. We further demonstrated that lapatinib-mediated suppression of proliferation in both cell lines was closely associated with remarkable inhibition of RTK signaling downstream of EGFR and erbB-2. We found that treatment with lapatinib for 24 h led to a dose-dependent suppression of EGFR, erbB-2, Akt, and Erk1/2 activation/phosphorylation in both cell lines (Fig. 1b-c). Lapatinib also induced a concurrent decrease in the expression of Cyclin D1, c-myc, and Bcl-2, which are critical regulators of cell proliferation and survival (Fig. 1b-c). These results are corroborated in other studies using human erbB-2-overexpressing breast cancer cell lines, including BT474, SKBR3, and MDA-MB-361 cells [21–24]. In particular, our results indicate that erbB-2-positive tumor cells derived from MMTV-erbB-2 transgenic mice were sensitive to lapatinib in vitro and the molecular signaling was specifically inhibited in accordance with erbB-2/EGFR-targeting therapeutics.

### Lapatinib inhibits in vivo tumor growth in the syngeneic tumor graft model using 78617 cells

**Fig. 2:**
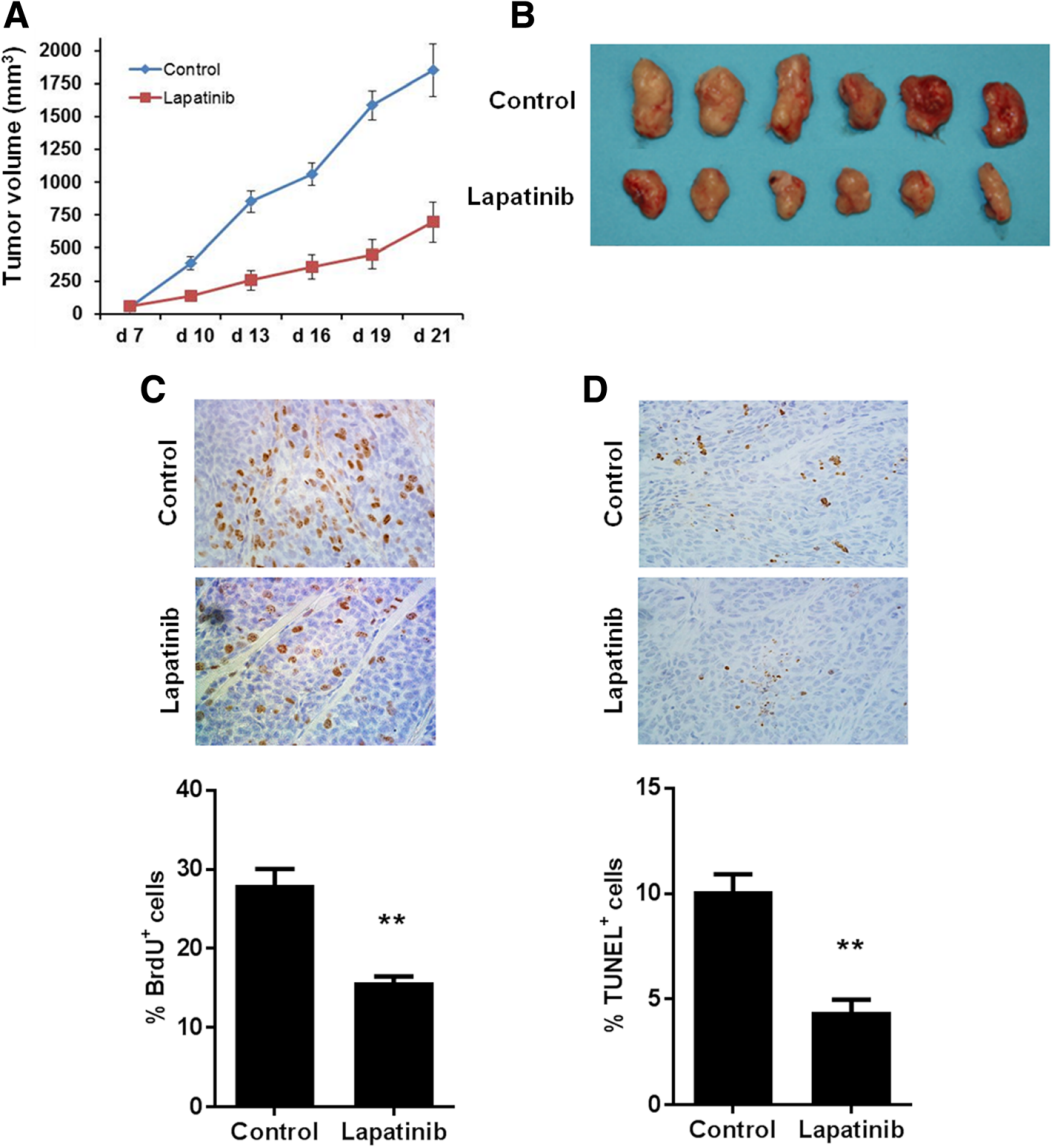
Lapatinib inhibits in vivo growth of syngeneic-grafted tumor cells. Eight-week-old MMTV-erbB-2 transgenic mice were subcutaneously injected with 1 × 10^6^ 78617 cells. The animals were then treated with 100 mg/kg/day lapatinib or vehicle control for 14 days. Tumor growth was monitored three times a week for three weeks. **a** Average tumor volumes in the control and lapatinib treatment groups are presented as means ± S.E. Images of syngeneic tumors after 14 days of lapatinib treatment are shown in (**b**). **c** Syngeneic tumor-grafted control and lapatinib (100 mg/kg/day for 14 days)-treated mice were injected with BrdU 90 min before euthanization Then tumors were excised and prepared for IHC analysis of BrdU nuclear incorporation. BrdU-positive cells are indicated by brown staining. **d** TUNEL staining of apoptotic cells is indicated by brown staining in tumor tissues from control and lapatinib-treated mice. BrdU-positive and TUNEL-positive cell percentages are graphed as the means ± S.E. (** *p* < 0.01)

To test the in vivo efficacy of lapatinib on tumor cells derived from MMTV-erbB-2 transgenic mice, we transplanted syngeneic 78617 cells into the flanks of female mice, followed by lapatinib (100 mg/kg/day) treatment for 14 days. As shown in Fig. 2a-b, lapatinib significantly inhibited tumor growth after 2 weeks of treatment. Multiple reports have also demonstrated that lapatinib abrogates tumor growth in xenograft mouse models using the BT474 human breast cancer cell line [25–28]. Consistently, when the harvested syngeneic tumors were stained for BrdU and TUNEL expression, we observed evident reduced cell proliferation and a decrease in apoptosis in the lapatinib-treated mice as compared to the vehicle-treated mice (Fig. 2c-d). Once more, these in vivo data provide further support to the notion that MMTV-erbB-2 mammary tumors are susceptible to lapatinib treatment.

### Short-term exposure to lapatinib during the risk window increases mammary tumor latency in MMTV-erbB-2 transgenic mice

**Fig. 3:**
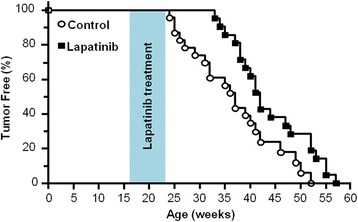
Short-term lapatinib exposure during the risk window increases mammary tumor latency in MMTV-erbB-2 transgenic mice. Mice were treated with lapatinib (100 mg/kg/day) from weeks 16-24 (8 weeks total). Mammary tumor latencies are presented with a Kaplan-Meier survival curve. The average latency for control and lapatinib groups were 37 and 42 weeks, respectively (*p* = 0.0154)

Based on our in vitro and syngeneic in vivo data, we tested the preventative efficacy of lapatinib in MMTV-erbB-2 transgenic mice using a novel strategy. Our goal was to develop a regimen that can achieve effective mammary tumor prevention with shorter exposure to lapatinib during the premalignant risk window. To this end, 16-week-old MMTV-erbB-2 mice were treated with lapatinib (100 mg/kg/day) for 8 weeks, which concluded at 24 weeks of age. This short-term exposure to lapatinib produced a dramatic delay in the onset of mammary tumors as compared to the control mice (Fig. 3). Distinctively, lapatinib-treated mice developed palpable tumors beginning at 33 weeks of age, while vehicle-treated mice developed tumors beginning at 25 weeks of age. This lapatinib-induced increase in tumor latency was maintained throughout the experiment, with the average latency for control and lapatinib groups being 37 and 42 weeks, respectively (*p* = 0.0154). Our data suggest that short-term, low dose exposure to lapatinib demonstrates efficacy in breast cancer prevention in genetically predisposed at-risk populations.

### Short-term exposure to lapatinib impedes mammary morphogenesis and suppresses cell proliferation

**Fig. 4:**
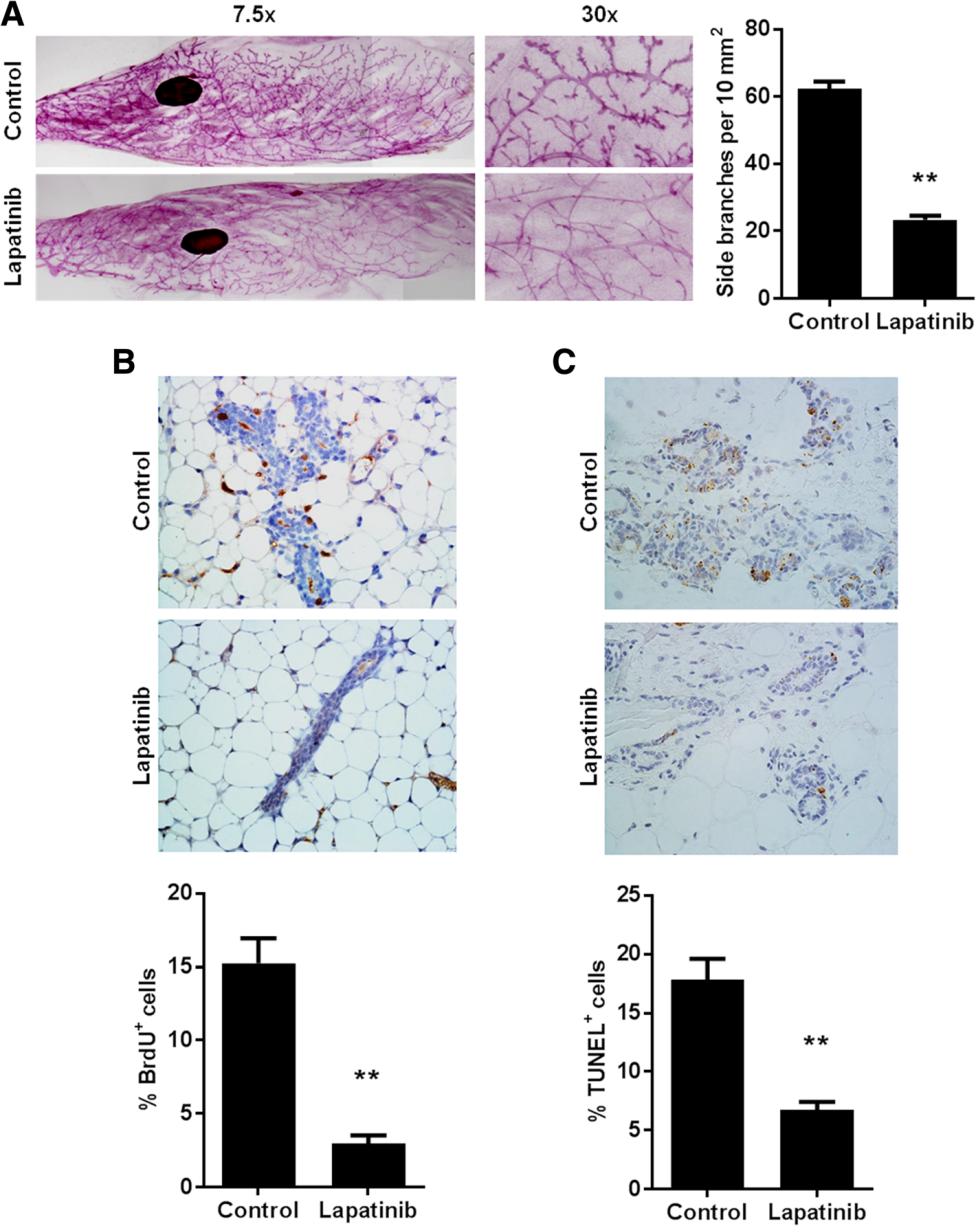
Lapatinib exposure impedes mammary morphogenesis and suppresses cell proliferation. **a** Mammary gland whole mounts were prepared from 24-week-old mice of control and lapatinib (100 mg/kg/day for 8 weeks)-treated groups. Representative images are shown at 7.5x and 30x magnification. The average number of side branches per 10 mm^2^ is graphed as the mean ± S.E. in the right panel (** *p* < 0.01). **b** Control and lapatinib (100 mg/kg/day for 8 weeks)-treated mice were injected with BrdU 90 min before euthanization at 24 weeks of age and then were prepared for IHC analysis of BrdU nuclear incorporation. BrdU-positive cells are indicated by brown staining. **c** TUNEL staining of apoptotic cells is indicated by brown staining in mammary gland tissues from 24-week-old vehicle and lapatinib-treated mice. BrdU-positive and TUNEL-positive cell percentages are graphed as the means ± S.E. (** *p* < 0.01)

Advanced mammary morphology is associated with breast cancer susceptibility in humans and is typically present in premalignant mammary tissues of MMTV-erbB-2 transgenic mice [29, 30]. To this end, we collected mammary glands of 24-week-old mice to examine the effects of lapatinib on breast tissue morphogenesis. As postulated, lapatinib (100 mg/kg/day) treatment for 8 weeks markedly inhibited mammary ductal growth and branching, as made evident by the decreased complexity of lateral branches and alveolar structures (Fig. 4a). These alterations demonstrate that lapatinib exposure has a profound impact on mammary ductal growth, which contributes to the delayed tumor onset in lapatinib-treated mice. To understand the cellular mechanisms underlying lapatinib-associated tumor inhibition and morphogenic reprogramming, we examined the proliferative status and apoptosis in mammary tissues from 24-week-old mice at the conclusion of lapatinib treatment using BrdU incorporation and TUNEL assays, respectively. As seen in Fig. 4b, lapatinib induced a striking decrease in the number of BrdU^+^ cells as compared to the control mammary gland samples, which is indicative of decreased cell proliferation. Interestingly, TUNEL assay data showed fewer apoptotic cells in the mammary gland tissues from lapatinib-treated mice as compared to the vehicle-treated mice (Fig. 4c). More apoptotic cells in the control tissues, especially in hyperplastic regions, could be explained by mitotic catastrophe in highly proliferative cells, which will be addressed in the Discussion section. Altogether, these data highlight the impact of short-term lapatinib exposure on mammary morphogenesis and suggest that lapatinib-mediated anti-proliferative effects may not only inhibit the growth of mammary epithelial cells, but also induce mammary reprogramming that contributes to long-term protection from mammary tumorigenesis even after drug withdrawal in MMTV-erbB-2 mice.

### Short-term lapatinib exposure inhibits RTK and ER signaling pathways in mammary tissues from MMTV-erbB-2 transgenic mice

**Fig. 5:**
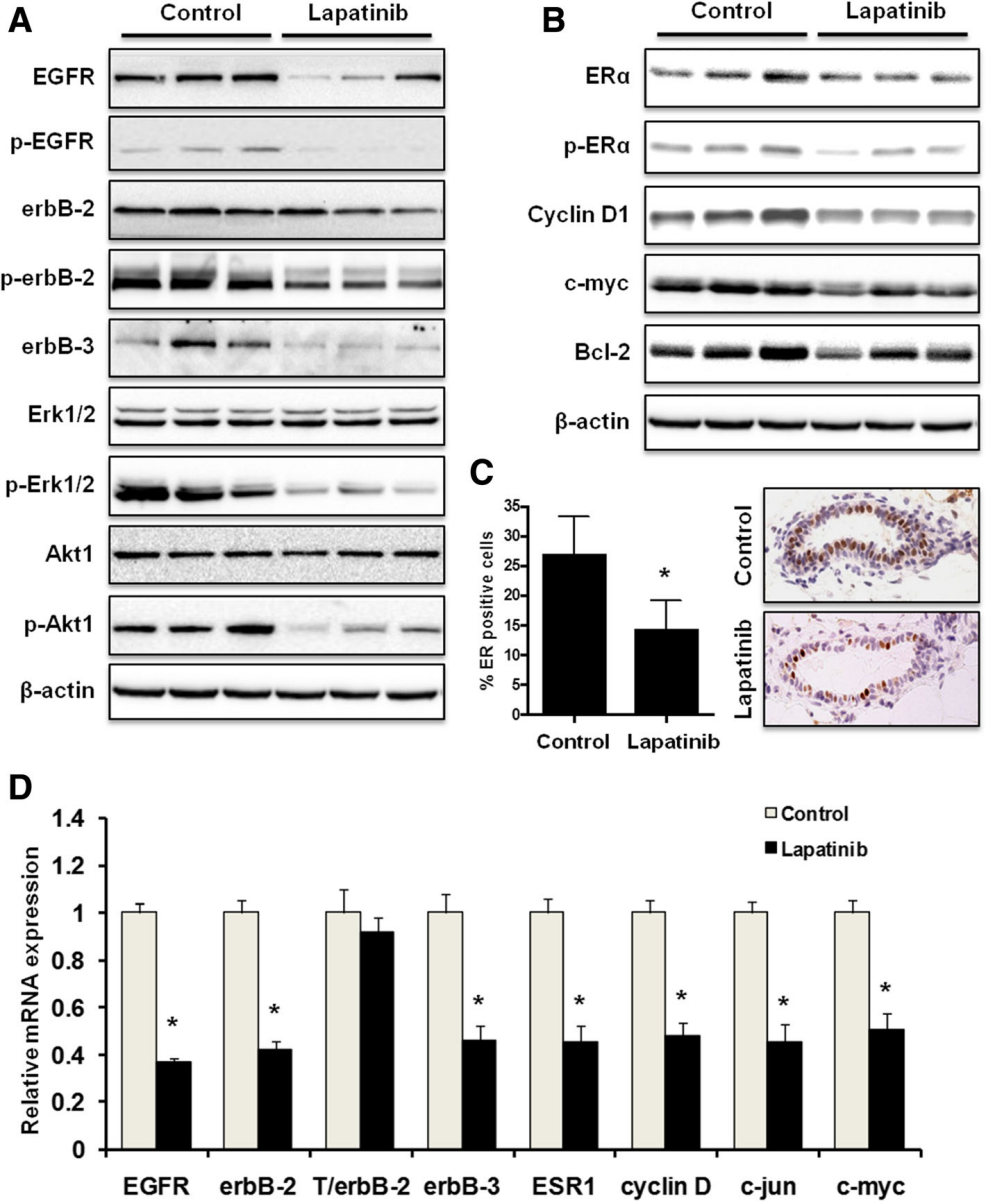
Lapatinib inhibits RTK and ER signaling pathways in vivo. **a, b** Total and phosphorylated protein levels of indicated markers in the mammary tissues (at 24 weeks of age) of mice from control and lapatinib (100 mg/kg/day for 8 weeks)-treated groups were detected using Western blotting. Protein samples from 3 mice in each group are shown. **c** Mammary tissue sections were prepared from 24-week-old control mice and mice treated with lapatinib (100 mg/kg/day) for 8 weeks. Percentages of ERα-positive cells were graphed as the means ± S.E. (* *p* < 0.05). Representative images of IHC analysis are shown with brown staining indicating ERα-positive cells. **d** mRNA levels of indicated markers in the mammary tissues of 24-week-old mice from control and lapatinib groups were quantified using real-time PCR. The relative mRNA expression of each indicated gene was graphed as means ± S.E. (* *p* < 0.05 versus the control for each gene)

To understand the molecular signaling in mammary tissues after short-term lapatinib treatment, we analyzed the protein expression and phosphorylation of key markers in the RTK and ER signaling pathways. As shown in Fig. 5a, lapatinib treatment induced a striking decrease in the protein levels of EGFR, p-EGFR, p-erbB-2, erbB-3, p-Erk1/2, and p-Akt1, indicating its effective regulation of growth factor activation and downstream PI3K/Akt and MAPK/Erk signaling. We found that both the expression and activation/phosphorylation of ERα were also significantly downregulated in a pattern similar to p-Akt1 and p-Erk1/2 downregulation. Consistently, protein levels of common ERα targets, including Cyclin D1, c-myc, and Bcl-2, were downregulated (Fig. 5b). In accordance with Western blot data, lapatinib significantly depleted the percentage of ER^+^ cells in these premalignant tissues as detected by IHC (Fig. 5c). To further understand the mechanistic functions of lapatinib, we explored the effects of lapatinib on mRNA expression of several genes involved in RTK and ER signaling in premalignant tissues. The results showed that lapatinib altered EGFR, erbB-2, and erbB-3 mRNA levels alongside ER gene expression (ESR1) and downstream targets Cyclin D1, c-jun, and c-myc (Fig. 5d). To note, T/erbB-2 (erbB-2 transgene) mRNA levels were not significantly affected by lapatinib treatment, suggesting that lapatinib does not interfere with MMTV-driven erbB-2 expression. Overall, these data demonstrate that lapatinib decreases tumor latency and mammary gland morphogenesis by negatively regulating the EGFR/erbB-2 and ERα signaling pathways. Moreover, concomitant alterations of EGFR/erbB-2 and ERα signaling suggest that lapatinib inhibits erbB-2-ER crosstalk in mammary tissues from MMTV-erbB-2 mice with short-term drug exposure.

### Lapatinib inhibits the stemness of erbB-2-overexpressing breast cancer in vitro

**Fig. 6:**
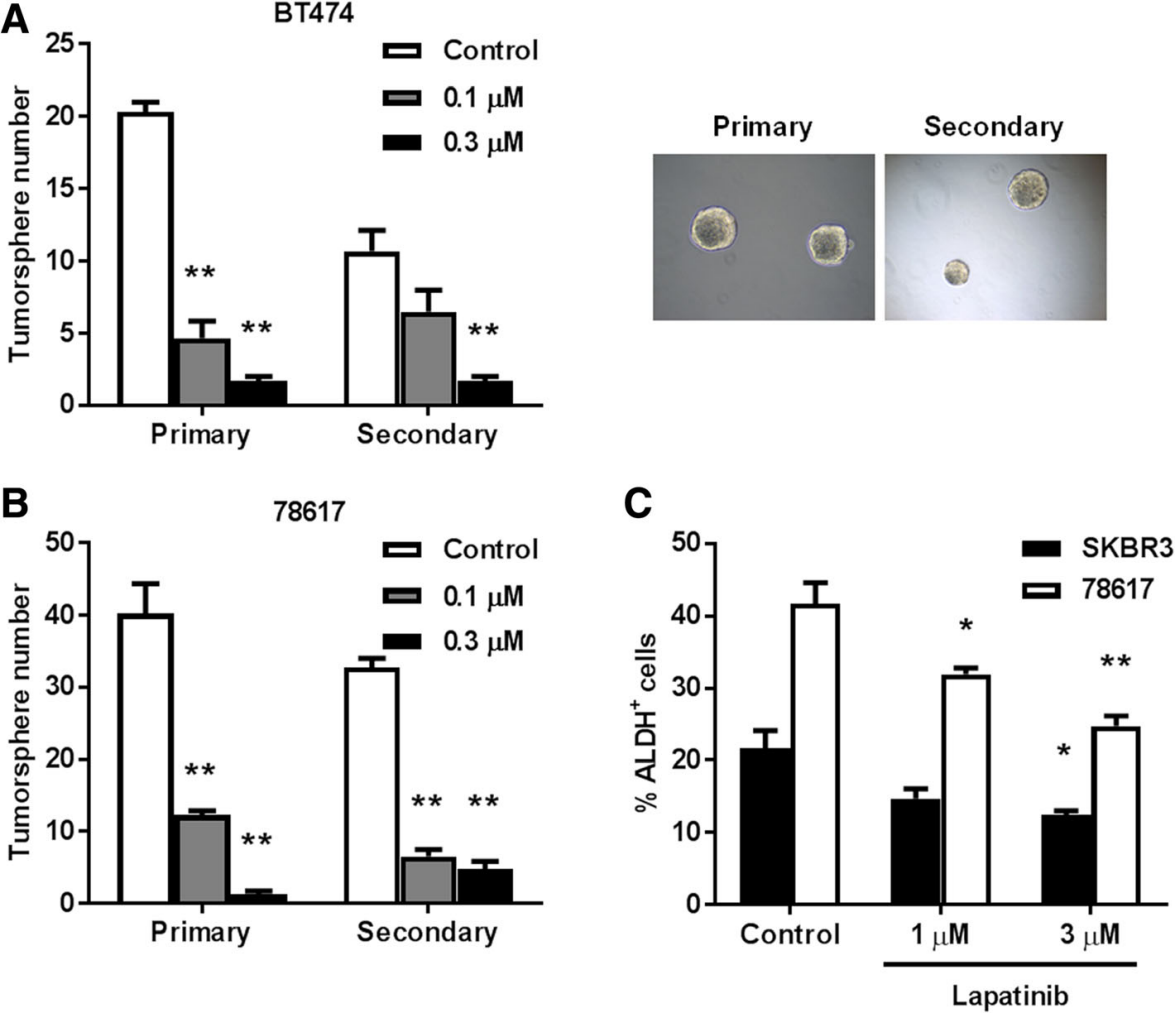
Lapatinib inhibits the stemness of erbB-2-overexpressing breast cancer in vitro**.** BT474 (**a**) and 78617 (**b**) cells were cultured in vitro and subjected to primary and secondary tumorsphere assays. In the primary tumorsphere assay, cells were initially treated with lapatinib (0, 0.1, or 0.3 μM) for 6 days for primary sphere formation. Then primary tumorspheres were harvested and replated for another 6 days under identical incubation conditions to form secondary spheres. Primary and secondary tumorsphere formations were recorded. Values are presented as the means ± S.E. (** *p* < 0.01 as compared to the corresponding untreated control samples). Representative images from each assay are depicted in the right panels. **c** SKBR3 and 78617 cells were treated with lapatinib (0, 1, or 3 μM) for 40 h, followed by quantification of ALDH-positive cells. The percentage of ALDH-positive cells was determined using the ALDEFLUOR detection kit with flow cytometry. Values are presented as the means ± S.E. (* *p* < 0.05; ** *p* < 0.01 as compared to the control for each cell line)

Previous reports have demonstrated that EGFR/erbB-2 and ER signaling can regulate mammary stem cells [31, 32]. To explain how short-term lapatinib exposure induced long-term protection from mammary tumor development, we postulated that the underlying mechanism involves lapatinib-mediated inhibition of tumor-initiating/cancer stem cells (CSCs). To this end, we investigated the consequences of lapatinib on in vitro cell lines with stem-like properties. Using tumorsphere assays, we demonstrated that exposure to low dose lapatinib inhibited both primary and secondary tumorsphere formation in erbB-2-overexpressing BT474 and 78617 cells, suggesting that lapatinib impairs the stemness and self-renewal capacity of these cells (Fig. 6a-b). We then investigated the effects of lapatinib on the ALDH^+^ cell populations in SKBR3, a classical ALDH-expressing cell line, and the mammary tumor-derived 78617 cells. As demonstrated in Fig. 6c, lapatinib significantly suppressed the percentage of ALDH^+^ cells, which is indicative of the CSC population, in both cell lines. Taken together, these results further provide evidence of the anti-cancer properties of lapatinib and suggest a possible mechanism of action for the long-lasting effects that we demonstrated with short-term exposure to lapatinib in MMTV-erbB-2 mice.

## Discussion

erbB-2-overexpressing breast cancers comprise nearly one-third of all breast cancer cases and are refractory to many therapeutics as drug-resistance often arises [4]. Therefore, the development of novel preventative strategies is crucial for women at a high-risk for this devastating disease. In this study, we explored a novel chemopreventative strategy using short-term, low dose lapatinib exposure to prevent mammary tumor development in MMTV-erbB-2 transgenic mice. We demonstrated that short-term lapatinib exposure during the premalignant risk window significantly delayed mammary tumor development, which was preceded by mammary gland growth inhibition in MMTV-erbB-2 mice. These growth inhibitory effects were associated with suppression of the MAPK/Erk pathway, PI3K/Akt pathway, and cell cycle progression (cyclin D1) in the premalignant mammary tissues in vivo, and the stemness of possible CSCs in vitro. Our results support the potential use of lapatinib as a chemopreventative agent and, in particular, provide proof of concept of short-term exposure to erbB-2/EGFR-targeting agents to achieve long-term protective effects.

Before in vivo testing of lapatinib-associated preventative effects, we examined the effects of lapatinib on 78617 and 85815 cells, two mammary tumor cell lines derived from MMTV-erbB-2 transgenic mice. Consistent with previous xenograft studies testing lapatinib-mediated effects on human breast cancer cell lines [21–28], data from our in vitro assays and syngeneic tumor models demonstrated that these tumor-derived cells were highly sensitive to lapatinib (Figs. 1 and 2). The doses used in our experiments were well-tolerated, which provides fundamental guidance for selecting the dose for our prevention study (Fig. 3). As a dual inhibitor of erbB-2 and EGFR, lapatinib also induced potent inhibition of the phosphorylation/activation of both RTKs, which led to remarkable suppression of PI3K/Akt and MAPK/Erk pathways, and other critical downstream regulators (Fig. 1). These sensitive biochemical and phenotypic changes not only demonstrate the therapeutic efficacy of lapatinib, but also support its potential as a chemopreventative agent.

Recently, small molecule inhibitors targeting RTKs have been investigated for their potential chemopreventative effects. Using the MMTV-erbB-2 mouse model, it has been shown that treatments with EGFR-targeting gefitinib or lapatinib both significantly inhibited the formation of premalignant lesions in the mammary glands and delayed spontaneous tumor development [19, 33]. In a recent clinical trial by DeCensi et al. (2011), lapatinib was administered to 60 women with erbB-2/Her2^+^ ductal carcinoma in situ (DCIS) for 3 weeks between the diagnostic biopsy and surgery [34]. Their results showed that this short-term lapatinib treatment decreased cell proliferation in ductal intraepithelial neoplasia (DIN), ductal hyperplasia without atypia (DH), and invasive erbB-2/Her2^+^ breast cancer. Data from both preclinical models and clinical trials support lapatinib as a preventative agent for women at risk for erbB-2/Her2^+^ breast cancers. While these advances provide the rationale for further clinical development exploiting the preventative capacity of lapatinib, more studies are needed to define optimal exposure timing treatment conditions.

Previously it was reported that treating MMTV-erbB-2 transgenic mice with 75 mg/kg BW lapatinib twice a day from 3 to 15 months of age resulted in inhibition of premalignant lesions and mammary tumor development [19]. In contrast to life-long (approximately 52 weeks) lapatinib exposure in MMTV-erbB-2 mice from the above study, we adopted the 8 week lapatinib treatment schedule to determine the preventative efficacy of a single daily dose of lapatinib (100 mg/kg/day) given for a short period of time during the risk window prior to tumor development. We demonstrated that this treatment regimen significantly delayed mammary tumor development by 5 weeks in MMTV-erbB-2 transgenic mice (Fig. 3). Specifically, the average tumor latency increased from 37 weeks in the vehicle-treated mice to 42 weeks in the lapatinib-treated mice after only a short treatment. Consistent with our hypothesis, short-term lapatinib exposure prior to tumor development induced long-lasting cancer preventative effects. Although the tumor inhibition observed in our study was not as impressive as the previous report by Strecker et al. [19] using long-term lapatinib treatment, the considerably shorter lapatinib treatment used in our study produced a substantial protective benefit. With further optimization of lapatinib doses and/or possible combination with other preventative agents, the outcomes of short-term lapatinib exposure could be greatly improved.

The significant lapatinib-induced changes in tumor latency that we demonstrate in Fig. 3 were also associated with substantial inhibition of epithelial cell proliferation and premalignant lesions (Fig. 4). In this regard, our morphogenic data showed that short-term lapatinib treatment resulted in striking changes in histopathological patterns and proliferative status, as indicated by decreases in ductal growth, lateral branching, epithelial density, and BrdU incorporation in mammary tissues from MMTV-erbB-2 mice (Fig. 4). These changes are consistent with the inhibition of erbB-2/EGFR signaling, which has a broad impact on ductal growth and morphogenesis in glandular and tumor development. Likewise, the alterations might be part of lapatinib-induced reprogramming that confers long-term protection from tumor development in this model. In the examination of apoptosis in the lapatinib-treated mammary gland tissues, we reported fewer TUNEL^+^ cells than in the control samples. This observation can be explained by highly proliferative cells in the tissues from vehicle-treated mice, which are associated with more apoptotic cell death through mitotic catastrophe and remarkable decreases in proliferative cells from lapatinib-treated mice [35, 36]. Although we did not see lapatinib-induced apoptosis at the given endpoint, we cannot rule out apoptosis induction during the initial, acute phase of lapatinib treatment. Our Western blot and PCR data indicated that short-term lapatinib exposure induces marked inhibition of erbB-2/EGFR signaling and downstream pathways/targets, which corroborates our in vitro results (Fig. 5). While these data confirm the connection between RTK inhibition and ultimate tumor prevention, the results also suggest that lapatinib may induce a broader impact on erbB-2/EGFR signaling beyond the canonical regulatory pathways. For instance, concurrent inhibition of erbB-2 and ER in mammary tissues suggests that lapatinib may block the crosstalk between these two signaling molecules, leading to downstream effects on multiple pathways. Together, our data indicate that the effects on morphogenesis and molecular signaling pathways are involved in the underlying mechanisms of short-term lapatinib exposure associated with mammary tumor prevention.

Recent progress in stem cell research indicates that mammary stem cells contribute to tumor heterogeneity, initiation, recurrence, and invasive potential through the differentiation into CSCs [37–40]. Due to the implication of CSCs in mammary tumor development and metastasis, stem cell-targeting therapies have been developed and are a proven therapeutic strategy [41–44]. In particular, numerous reports have shown that metformin selectively inhibits CSCs in erbB-2^+^ breast cancer models [42–44]. To this end, we examined stemness in erbB-2-overexpressing tumor-derived cells and cell lines (Fig. 6). Our findings indicated that lapatinib inhibits tumorsphere stemness and self-renewal, which is represented by decreased primary and secondary tumorsphere numbers after lapatinib treatment. ALDH1 activity/overexpression is correlated to CSCs and poor prognosis in various tissue-specific cell types, including mammary epithelial cells [45–47]. As such, we demonstrated that lapatinib suppressed ALDH1 activity in 78617 mammary tumor-derived cells and BT474 breast cancer cells. Overall, our data indicate that lapatinib inhibits CSC-like properties in vitro, providing a plausible mechanism for the anti-tumorigenic effects that we report in vivo.

## Conclusions

In conclusion, short-term exposure to lapatinib during the premalignant risk window for developing erbB-2-overexpressing mammary tumors resulted in a striking shift in tumor onset, which was preceded by delayed mammary morphogenesis and inhibition of molecular signaling pathways that regulate cell proliferation, cell survival, and stem cell self-renewal. Our study suggests that short-term, low dose lapatinib exposure during the premalignant phase of breast cancer development is a promising strategy to prevent the development of erbB-2-overexpressing mammary tumors. A short-term, low dose chemoprevention protocol not only improves patient compliance, but also minimizes chronic side effects, making short-term lapatinib exposure a potentially ideal preventative strategy with long-lasting outcomes.

## Abbreviations

ALDH1: Aldehyde dehydrogenase 1
ATCC: American type culture collection
BrdU: 5-bromo-2’-deoxyuridine
CSCs: Cancer stem cells
DAB: Diaminobenzidine
DCIS: Ductal carcinoma in situ
DEAB: Diethylaminobenzaldehyde
DH: Ductal hyperplasia without atypia
DIN: Ductal intraepithelial neoplasia
EGFR: Epidermal growth factor receptor
ER: Estrogen receptor
FBS: Fetal bovine serum
HRP: Horseradish peroxidase
IHC: Immunohistochemistry
MAPK: Mitogen-activated protein kinase
MMTV: Mouse mammary tumor virus
PI3K: Phosphatidylinositol 3-kinase
RTK: Receptor tyrosine kinase
RT-PCR: Real-Time PCR
TUNEL: Terminal deoxynucleotidyl transferase-mediated dUTP nick end labeling
